# A sensitive and less cytotoxic assay for identification of proliferating T cells based on bioorthogonally-functionalized uridine analogue

**DOI:** 10.1016/j.jim.2022.113228

**Published:** 2022-01-21

**Authors:** F.C. Stempels, A.S. de Wit, M.S. Swierstra, S. Maassen, F. Bianchi, G. van den Bogaart, M.V. Baranov

**Affiliations:** Department of Molecular Immunology and Microbiology, Groningen Biomolecular Sciences and Biotechnology Institute, University of Groningen, Groningen, the Netherlands

**Keywords:** T cell activation, T cell proliferation, Ethynyl-uridine, EdU, Dilution-based probe

## Abstract

Quantitative detection of T cell proliferation is an important readout in immunology research, as it is one of the hallmarks of T cell activation. Fluorescence-based methods for T cell proliferation mostly rely on the usage of probes that non-specifically conjugate to free primary amine groups in cells. Each cell division then results in a two-fold dilution of the probes which is detectable with flow cytometry. However, questions have been raised about cytotoxicity of these dilution-based T cell proliferation probes and they potentially affect T cell activation. An alternative assay relies on the incorporation of the uridine analog BrdU in the DNA of dividing T cells that can be detected with an antibody, but this requires harsh fixation and denaturation conditions. Recently, a new assay for detection of cell proliferation has been developed, based on the incorporation of the bioorthogonally-functionalized uridine analog 5-ethynyl-2’ -deoxyuridine (EdU). Goal of this study was to compare the sensitivity and cytotoxicity of the EdU assay with a widely-used dilution-based T cell proliferation probe, CellTrace Far Red. We found that, compared to the dilution-based probe, the EdU-based assay better preserves T cell viability, is more sensitive for detecting T cell proliferation, and allows for better discernable interferon gamma responses.

## 1 Introduction

Cell proliferation is an important parameter in immunology as it is a measure for the activation of B lymphocytes (Hodgkin et al., 1996) and T lymphocytes (Hasbold et al., 1998; Marchingo et al., 2016), and also for hematopoietic stem cells (Oostendorp et al., 2000). The potency of dendritic cells (DCs) to induce T cell proliferation is commonly used for the evaluation of DC function, thereby playing an important role in the development of new DC-based therapies (Tel and de Vries, 2012; Tel et al., 2012). In addition, by monitoring patient-specific immune responses, cell proliferation assays could aid in predicting therapy outcomes and thereby play a central role in the development of personalized medicine. For example, in PDL1-expressing non-small-cell lung carcinoma, T cell proliferation has been used to predict patientspecific anti-PD1 responses (Kagamu et al., 2020). Due to the plethora of applications, fast and sensitive methods for detecting proliferating T cells are in demand, both for fundamental and translational immunology.

T cell proliferation takes place as a result of T cell activation. T cells are activated by antigen presenting-cells (APCs) like DCs and macrophages. For this activation, an APC harboring peptide-MHC complexes is recognized by the T cell receptor (TCR), flanked by its co-receptors CD3 and CD4 or CD8. In addition, some APCs express costimulatory molecules CD80 and CD86 on their surface, which both bind to the T cell surface glycoprotein CD28 required for activation of naive T cells (Howland et al., 2000). An additional costimulatory molecule on the surface of APCs, CD40, binds to CD40 ligand (CD40L) on the surface of activated T cells, ensuring sustained T cell responses (Howland et al., 2000). CD40L is expressed early by activated T cells and can thus function as an early activation marker. CD40-CD40L binding allows bidirectional communication between the APCs and T cells, as CD40L also induces effector functions in APCs. For example, CD40 stimulation activates DCs and induces their production of the pro-inflammatory cytokine interleukin (IL)-12, a process called DC licensing (Cella et al., 1996; Stüber et al., 1996). In B cells, CD40 stimulation leads to cell differentiation and isotype switching in the germinal center reaction (Daoussis et al., 2004; Hasbold et al., 1998). Activated T cells secrete IL- 2 at earlier division stages (Sojka et al., 2004) and interferon γ (IFN-γ) at later divisions (Hasbold et al., 1999).

A standard method for cell proliferation analysis uses carboxyfluorescein succinimidyl ester (CFSE) (Lyons and Parish, 1994). This method is often used for the analysis of T cell proliferation (Di Carluccio et al., 2019; Hasbold et al., 1999). The cell permeable CFSE dye covalently binds to the cell surface and intracellular amines via its succinimidyl ester moiety. Cells are loaded with CFSE at the start of incubation and each round of cell division reduces the number of fluorophores per cell by 50%, as the dye is equally distributed over the daughter cells. Thereby, CFSE facilitates cell division analysis by dye dilution (Filby et al., 2015). However, CFSE labelling can reduce T cell proliferation, viability, and responsiveness, and requires specific buffering conditions to minimize its toxicity (Ten Brinke et al., 2017; Hasbold et al., 1999; Lašt’ovička et al., 2009; Parish et al., 2009; Quah et al., 2007). Cell-Trace, an improved CFSE alternative engineered to overcome these issues, is less toxic and provides higher fluorescence brightness allowing for the determination of more rounds of cell division (Filby et al., 2015; Quah and Parish, 2012). In addition, CellTrace is available in multiple colors, making it compatible with fluorescently labeled antibodies (Tempany et al., 2018). However, the CellTrace probes still affect the cell division program and reduce the number of proliferative cells (Filby et al., 2015; Tempany et al., 2018).

Another limitation of CellTrace and other dilution-based T cell proliferation probes is that cell division can only be determined after completion of a full round of cell division. Therefore, T cells that are still in the process of division cannot be identified with these dilution-based probes. Alternatives to dilution-based T cell proliferation probes, which do allow for the detection of actively dividing cells, are DNA-binding probes and detectable nucleoside analogues. DNA-binding probes, including propidium iodide and DRAQ5, facilitate to differentiate haploid (G1-phase) and diploid cells (G2-phase of cell division) (Scheipers and Reiser, 1998; Yuan et al., 2004). However, the difference between fluorescence intensities between those cell populations is only 2-fold and there are cells with intermediate fluorescence signals from cells in the process of DNA replication, making it challenging to differentiate these populations. Compared to such DNA-binding probes, detectable nucleosides have better signal to background ratios. These become incorporated in the newly-synthesized DNA during S-phase (Romar et al., 2016). Since the cells only have to be exposed to this reagent for several hours for detectable incorporation, no complete cell division is required. Often, radiolabeled tritiated thymidine is used as detectable nucleoside and the level of incorporated thymidine is measured by total radioactivity. While T cell proliferation measured with tritiated thymidine correlates well with dilution-based probes (Di Blasi et al., 2020; Ten Brinke et al., 2017), this assay does not provide information about individual cells and has low sensitivity (Main and Walwick, 1961).

A nonradioactive nucleoside analogue is 5-bromo-2’ -deoxyuridine (BrdU), which can be detected by an antibody (Gratzner, 1982). Because BrdU is incorporated into DNA, a brief pulse can indicate the number of cells in S phase of the cell division cycle (Hasbold et al., 1999). This method has been used for assessing T cell proliferation (Messele et al., 2000; Rufer Marco Migliaccio and Miguel Sousa Alves, 2021). However, the detection of BrdU requires denaturation of double-stranded DNA by either acid, heat or nuclease treatment, which not only elongates the protocol but also destructs most epitopes, hampering subsequent immunolabeling.

A new approach for detecting cell division involves the use of bio-orthogonal 5-ethynyl-2’-deoxyuridine (EdU), a molecule chemically related to BrdU (Salic and Mitchison, 2008). A copper(I)-catalyzed bioorthogonal chemistry reaction (Rostovtsev et al., 2002) enables the covalent binding of azide-conjugated fluorophores to the ethynyl group of EdU. Therefore, the detection of EdU does not require denaturation or antibodies and is compatible with subsequent immunolabeling. A comparison of BrdU and EdU showed comparable results in proliferation of cell lines (Diermeier-Daucher et al., 2009). EdU has also been used to measure T cell proliferation both *in vitro* and *in vivo* (Alvarez et al., 2020; Poujol et al., 2014; Sun et al., 2016; Sun et al., 2012; Vibert and Thomas-Vaslin, 2017; Yu et al., 2009). However, the sensitivity of EdU and its effects on human T cell proliferation and activation have not yet been directly compared with dilution-based T cell proliferation probes.

In this study, we side-by-side compared EdU with the dilution-based probe CellTrace Far Red, which is a standard in the field for T cell proliferation. We show that the EdU bioorthogonal chemistry-based approach requires significantly less time, better preserves cell viability, provides high sensitivity for dividing T cells, and shows better discernable IFN-γ responses.

## 2 Methods

### 2.1 PBL and moDC generation

Peripheral-blood mononuclear cells (PBMCs) were isolated by density gradient centrifugation from buffy coats of 4 healthy donors as described previously (Baranov et al., 2019). Approval to conduct experiments with human blood samples was obtained from the blood bank and all experiments were conducted according to national and institutional guidelines. Informed consent was obtained from all blood donors by the Dutch blood bank. Samples were anonymized and none of the investigators could ascertain the identity of the blood donors. In brief, the buffy coat was mixed with diluting solution (PBS supplemented with 0.4% 0.5 M EDTA) in a 1:1.4 ratio, and subsequently added to SepMate-50 tubes (Stemcell Technologies) filled with 15 ml ficoll. The tubes were centrifuged (1250 rcf, 15 min, 21 °C), and the top layers (containing serum and PBMCs) were collected into new 50 ml tubes. Diluting solution was added to reach a total volume of 50 ml. The cells were spun down (485 rcf, 10 min, 21 °C), the supernatant was discarded, and the cell pellet resuspended in 50 ml wash buffer (PBS supplemented with 0.4% 0.5 M EDTA and 1:100 10% BSA). The cells were again centrifuged (437 rcf, 5 min, 4 °C), and the pellet resuspended in 50 ml wash buffer. This process was repeated until the supernatant became clear. The cells were then counted and washed once with RPMI serum free medium (Gibco).

Monocyte-derived dendritic cells (moDCs) and peripheral blood leukocytes (PBLs) were isolated from the PBMCs as described previously (Baranov et al., 2019). Briefly, PBMCs were seeded in a T75 culture flask (Corning Costar) in a concentration of 1.25 × 10^7^ cells/ml in 8 ml of RPMI medium supplemented with 2% human serum and 2 mM _L_-glutamine. After incubation of 1 h at 37 °C and 5% CO_2_, the medium (containing the nonadherent lymphocytes) was transferred to 50 ml tubes. This PBL faction was then frozen down for storage (see below). The T75 culture flasks containing the adherent monocytes were washed extensively with two rounds of adding 6 ml PBS, followed by tapping the flasks to remove loosely adherent lymphocytes. Subsequently, another two rounds of 6 ml PBS washes were performed, this time without tapping. The cells were then cultured for 6 days at 37 °C and 5% CO_2_ in 8 ml RPMI medium supplemented with 300 U/ml IL-4, 450 U/ml GM-CSF, 1% antibiotic-antimycotic, 2 mM _L_-glutamine, and 10% fetal calf serum (FCS) (RPMI-FCS-L-Glu-AA medium). At day 3, 4 ml complete RPMI medium containing 900 U/ml IL-4, 1350 U/ml GM-CSF, and 10% FCS was added to each flask. On day 6, immature moDCs were harvested. The medium was collected, and the PBS of one ice-cold PBS wash was added to the collected medium. Next, moDCs were detached from the flask by incubating the cells in the fridge for 1 h with 6 ml ice-cold PBS. This cell suspension was then also pooled with the collected medium and spun down (437 rcf, 5 min, 4 °C). The cell pellet was resuspended in RPMI medium.

### 2.2 Cryopreservation of PBLs and moDCs

The isolated PBLs and moDCs cell suspensions were frozen down in cryo-vials containing 1 ml of 7 × 10^6^ cells and 2.5 × 10^6^ cells respectively in 10% DMSO and 40% FBS. The cells were first frozen in cryogenic vials (Greiner) using a 1 °C controlled-rate cell freezing container (Corning Coolcell LX) which was put at -80 °C for 24 h. Subsequently, the vials were placed in liquid nitrogen for long-term storage.

### 2.3 MLR reaction and bead stimulation

Cryopreserved PBLs and moDCs of 4 donors were thawed, washed twice with PBS, pelleted at 300 xg for 5 min in 15 ml, and resuspended at 1 × 10^5^ (PBLs) and 1 × 10^4^ (moDCs) cells / 50 μl in RPMI medium supplemented with 10% decomplemented human serum, 2 mM _L_-glutamine, and 1% antibiotic-antimycotic (RPMI-HS-L-Glu-AA medium). The allogeneic cocultures were made by mixing moDCs and PBLs from different donors in a 1:10 ratio in 100 μl in a 96-well non-adherent suspension culture plate. Mixtures of moDCs and PBLs from the same donor were used as negative control for T cell activation. As an additional negative control, 50 μl of PBLs and 50 μl moDCs of each donor was mixed with 50 μl of just RPMI-HS-L-Glu-AA medium. As a positive control, 50 μl of PBLs of each donor were mixed with 50 μl anti-CD3 anti-CD28 beads (Gibco, 11131D, Human T-Activator CD3/CD28 Dyna-Beads) in a ratio of 1 bead per 25 cells. Two identical 96-well plates with co-cultures were made in the above-described manner, one for doing a EdU assay and the other for doing a CellTrace-assay. For the CellTrace-assay plate, the PBLs were first stained with CellTrace (see below) before being mixed with moDCs or DynaBeads. All co-cultures were incubated for 5 days at 37 °C and 5% CO_2_.

### 2.4 CellTrace Far Red loading

On day 0, before mixing the PBLs with moDCs or anti-CD3 anti-CD28 beads, 1 × 10^6^ cells/ml of PBLs per donor were pelleted (432 rcf, 5 min) and resuspended in CellTrace Far Red staining solution (Invitrogen C34564, 1:1000 in PBS) and incubated for 20 min at 37 °C. Afterwards, to remove any free dye in the solution, RPMI-FCS-L-Glu-AA medium was added at a volume of 5 times the original staining volume. The cells were spun down again, and the pellet was resuspended at 1 × 10^5^ cells/50 μl in RPMI medium with 10% FCS. Subsequently, the CellTrace labeled PBLs were mixed with the moDC or beads and cultured for 5 days in RPMI-HS-L-Glu-AA medium as described above.

### 2.5 EdU incorporation and detection

EdU (Component E from the Baseclick GmbH, ClickTech EdU T cell proliferation kit 488; BCK-TCell-FC488) was added to the MLR or bead-stimulated PBLs cultures at a 10 μM final concentration on day 5, 2 h before harvesting the cells unless indicated otherwise. For harvesting, the 96-well plate containing the allogeneic and autologous cocultures and controls was centrifuged (432 rcf, 3 min, 21 °C). The clarified supernatants were collected and frozen before cytokine detection by ELISA. The cell pellets were resuspended and washed with 150 μl PBS. At this point, live/dead staining with eFluor780 and APC-conjugated antibody staining was performed (see below). Before the bio-orthogonal reaction, the cells were washed once with 100 μl PBS containing 1% BSA, and subsequently fixed with 100 μl 4% paraformaldehyde (PFA) at room temperature in the dark for 15 min. Afterwards, the cells were washed twice with 150 μl PBS containing 1% BSA and incubated for 20 min on ice with 100 μl 10% saponin in PBS containing 1% BSA buffer (component P from the BCK-TCell-FC488 kit). The cells were then incubated for 1 h with 10 μl of the BCK-TCell-FC488 click assay cocktail, which contained deionized water, reaction buffer (1:10), reactor solution (1:25), Eterneon^2^ GREEN 488 dye azide (1:100; 6-FAM Azide alternative), and buffer additive (1:10) (components RB, C, D, and B from the BCK-TCell-FC488 kit). We also performed experiments with Eterneon^2^ YELLOW dye azide (Baseclick GmbH, 5-TAMRA azide alternative) and Eterneon^2^ RED dye azide Baseclick GmbH, Cy5 alternative) instead of Eterneon^2^ GREEN dye azide. After incubation, the cells were washed with 200 μl saponin buffer from the kit. All steps were performed according to the manufacturer’s instructions.

### 2.6 Antibody staining and flow cytometry

Before the fixation step, all cell samples were stained with Fixable Viability Dye eFluor780 (eBioscience, 1:2000 in cold PBS, 100 μl) on ice for 10 min followed by 100 μl/per well wash with PBS containing 1% BSA. The EdU loaded cells were stained for the CD3 receptor with 25 μl mouse anti-CD3 APC-conjugated antibody (Invitrogen, 1:100 in 1% BSA) on ice for 30 min. This staining was not performed with the Cell-Trace samples, since CellTrace signal is detected in the APC spectrum. After washing with 100 μl PBS containing 1% BSA, the fixation, permeabilization and click reaction were continued as described above. After the click reaction and saponin buffer wash, both the EdU and CellTrace samples were stained on ice for 30 min with mouse anti- CD40L PE-conjugated antibody (Clone TRAP-1, Beckman, IM2216U, 1:100 in 1% BSA) at 25 μl/well, and the CellTrace samples were stained with mouse anti-CD3 BV421-conjugated antibody (Clone SK7, BD Horizon, 563797, 1:100 in 1% BSA) in 25 μl/well. The PE staining was performed after the EdU detection reaction as recommended by the protocol of the BCK-TCell-FC488 Kit. The cells were washed once with 100 μl/well saponin buffer (component P from the BCK-TCell-FC488 kit) and once with 100 μl/well PBS. Finally, the cells were resuspended in 100 μl/well PBS and analyzed with flow cytometry.

Flow cytometry was performed using a CytoFlex S flow cytometer (Beckman Coulter Inc., Indianapolis, IN, USA), and the data was analyzed using FlowJo software. For both the EdU and CellTrace samples, the lymphocyte population was gated by using forward and side-ward scatter (FSC; SSC), and the single cells were selected by plotting forward scatter area against forward scatter height (FSC-A; FSC-H). An average of approximately 1 × 10^4^ total events were analyzed after exclusion of dead cells by gating on APC-A750^−^ (eFluor780 negative) cells. For the EdU samples, activated T cells were selected by first gating on CD3^+^ (CD3-APC^+^) cells, and subsequently analyzing the percentage of CD40L^+^ cells by gating on CD40L-PE. Proliferating T cells were selected by detecting EdU incorporation. This was done by gating on the CD3^+^ (CD3-APC^+^) population and analyzing the percentages of FITC-A^+^ (EdU Click Eterneon GREEN^+^) cell). For the CellTrace samples, living and dividing cells were selected by first gating on APC-A750^−^ and subsequently plotting percentages of low APC-A^+^ (i.e., decreased signal of CellTrace Far Red) cells.

### 2.7 Confocal microscopy

The cells were first cultured in suspension 96-well plate as described in the section MLR reaction and bead stimulation. CellTrace-treatment was done on day 0. On day 4 the cells were collected from 96-well plates and transferred to 8-well glass bottom plates coated with polylysine (Ibidi USA, 80824) (for 2 h) and subsequently loaded with EdU. The rest of the click-reaction protocol is described in the section EdU incorporation and detection. Cell fixation with 4% PFA was followed by two PBS washes. The cells were kept in PBS during imaging. Samples were imaged with a Zeiss LSM 800 confocal microscope fitted with a 40 × 1.4 NA air objective.

### 2.8 Cytokine secretion detection with ELISA

The levels of IFN-γ and IL-2 secretion were detected using IFN-γ (ThermoFisher Invitrogen, 88-7316-88) and IL-2 (ThermoFisher Invitrogen, 88-7025-88) human uncoated ELISA kits. High-affinity protein binding ELISA plates (ThermoFisher) were coated overnight at 4 °C with 50 μl/well of capture antibody (pre-titrated, purified anti-human IFN-γ or IL-2 antibody, 1:250 in coating buffer). The plates were washed 5 times with 150 μl/well washing buffer (PBS, 0.05% Tween-20), blocked for 1 h at room temperature with 100 μl blocking buffer (ELISA/ELI-SPOT diluent diluted 1:5 in MilliQ water), and again washed 3 times with 150 μl/well washing buffer. Afterwards the samples and standard curves were prepared in the appropriate wells. The sample supernatants were thawed on ice and added 10 μl/well to 40 μl/well of blocking buffer. For the standard curve, a 2-fold serial dilution in blocking buffer for 8 points and a blank condition was made from the standard (500 pg/ml recombinant human IFN-γ or 250 pg/ml recombinant human IL-2). The plate was incubated overnight at 4 °C. The next day the plate was washed 10 times with 150 μl/well washing buffer, and subsequently incubated for 1 h at room temperature with 50 μl/well detection anti-body (pre-titrated, biotin-conjugated anti human IFN-γ or IL-2 antibody, 1:250 in blocking buffer). After another 10 washes with 150 μl/well washing buffer, the plate was incubated for 30 min at room temperature with 50 μl/well of diluted streptavidin-horseradish peroxidase (HRP; 1:250 in blocking buffer). The plate was again washed 10 times with 150 μl/well washing buffer and subsequently incubated with 100 μl/well 1× 3,3’,5,5’-tetramethylbenzidine (TMB) solution for approximately 15 min at room temperature in the dark. Lastly, 50 μl/well of Stop Solution (1 M H_3_PO_4_) was added. The colour reaction’s absorbance values for TMB were measured at 450 nm in a Biotek Synergy HTX plate reader. The background was measured at 570 nm and subtracted from the 450 nm values. The data was processed and analyzed using Microsoft Excel and Graphpad Prism.

## 3 Results

### 3.1 EdU-based protocol preserves T cell viability

**Fig. 1:**
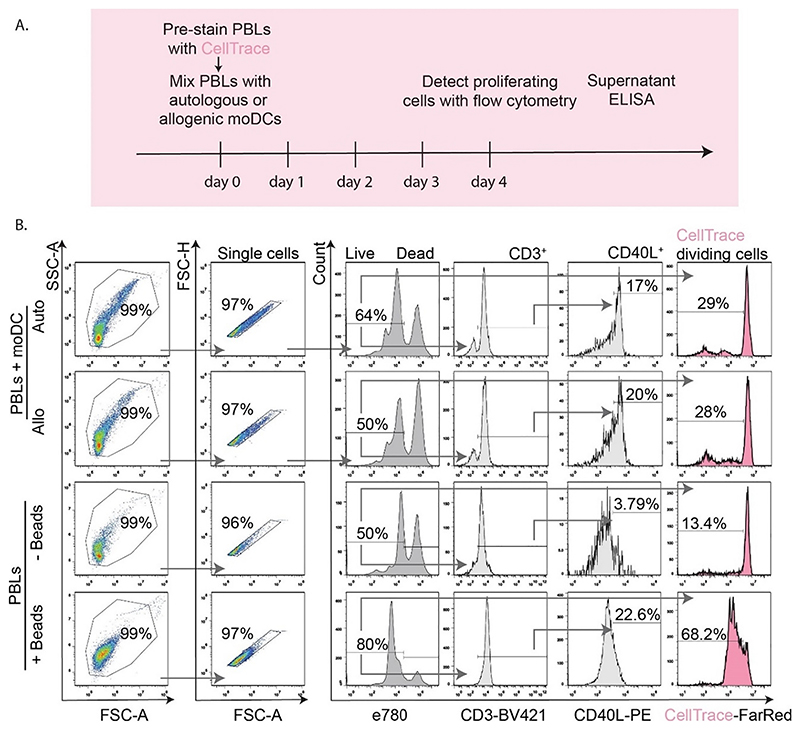
CellTrace detection of proliferated T cells and flow cytometry gating. A. Scheme of mixed leukocyte reaction (MLR) with CellTrace pre-loading of PBLs on day 0 prior to co-culturing with moDCs. MoDCs and PBLs from the same donor (autologous, Auto) pairs were used as negative controls for T cell activation. Pairs with PBLs and moDCs from different donors (allogenic, Allo), were used to initiate T cell proliferation/activation, which was detected by flow cytometry and ELISA. B. Flow cytometry gating strategy used for the MLRs or for the PBLs activated with anti-CD3 anti-CD28 beads (+/-Beads). The cells were selected based on forward and side scatter, followed by exclusion of cell clumps (single cells). Next, eFluor780 staining was used to exclude dead cells. Live cells were analyzed for CD3, CD40L and CellTrace. A reduction of intensity in the CellTrace histograms indicates proliferated cells. Arrows show gating order. Experiments were performed with cells isolated from 4 donors; quantification in Fig. 3.

**Fig. 2:**
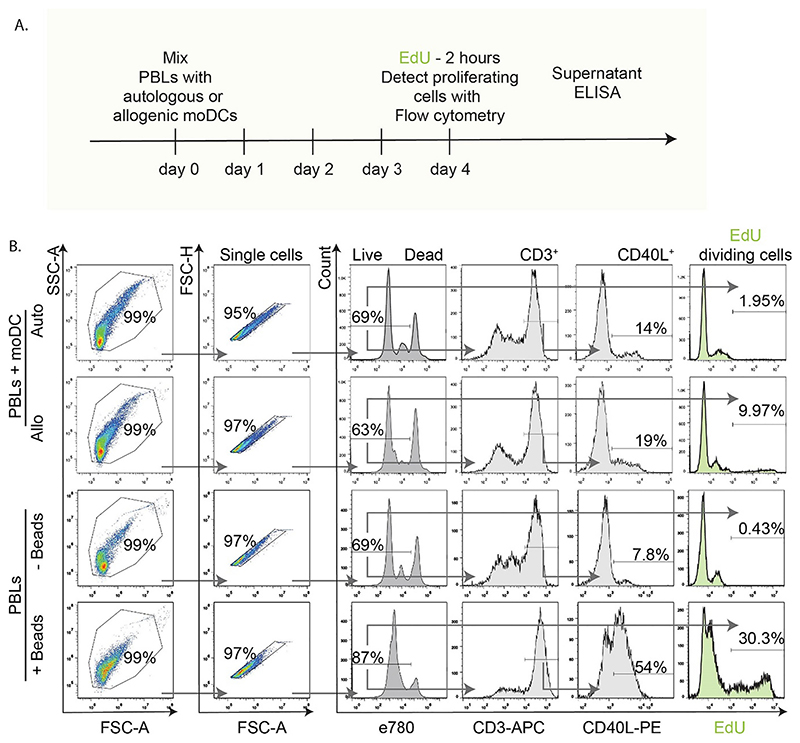
EdU-based detection of proliferating T cells and flow cytometry gating. A. Scheme of MLR experiments as in Fig. 1A, with the difference that cell loading with EdU was performed on day 4; two hours prior to fixation and analysis. B. Flow cytometry gating strategy used for the MLR with autologous (Auto) or allogenic (Allo) pairs of PBLs and moDCs or for the PBLs activated with anti-CD3 anti-CD28 beads (+/-Beads). Cells were selected based on forward and side scatter, followed by exclusion of cell clumps (single cells). Next, dead cells were excluded by selecting cells with low eFluor780 intensity using the same gate as for the CellTrace samples. Live cells were analyzed for CD3, CD40L and EdU incorporation (detected by bioorthogonal click-chemistry). An EdU histogram intensity shift towards the right indicates proliferating cells. Arrows show gating order. Experiments were performed with cells isolated from 4 donors; quantification in Fig. 3.

We tested EdU incorporation in two protocols for stimulation of human T cell activation, and compared this side-by-side with the dilution-based T cell proliferation probe CellTrace Far Red. Peripheral blood leukocytes (PBLs) were isolated from human blood buffy coats of 4 donors. From the same buffy coats, immature monocyte-derived dendritic cells (moDCs) were generated. The moDCs and PBLs were then co-cultured (Figs. 1A and 2A), creating either autologous pairs (i.e., moDCs and PBLs from the same donor) which do not induce T cell activation, or allogenic pairs (moDCs and PBLs from different donors) which induce a mixed leukocyte reaction (MLR). These co-culturing experiments were performed for 5 days. The MLR is a canonical assay to study the capacity of DCs to activate T cells in a host-vs-graft response (Trumpfheller et al., 2021). The PBLs were loaded with CellTrace before they were mixed with the moDCs (Fig. 1A). In contrast, EdU was added to the complete PBL-moDC cell mixture on the final day and incubated with the cells for just 2 h (Fig. 2A).

As a second assay for T cell activation, PBLs were stimulated for 5 days with 2–3 μm-sized beads carrying antibodies directed against CD3 and CD28 on their surface. These so-called DynaBeads simultaneously activate both the CD3 and CD28 receptors and thereby induce strong T cell activation. Similar to the MLR experiments, the PBLs were either pre-loaded with CellTrace prior to addition of the beads, or with EdU during the final 2 h of the incubation on day 4.

**Fig. 3:**
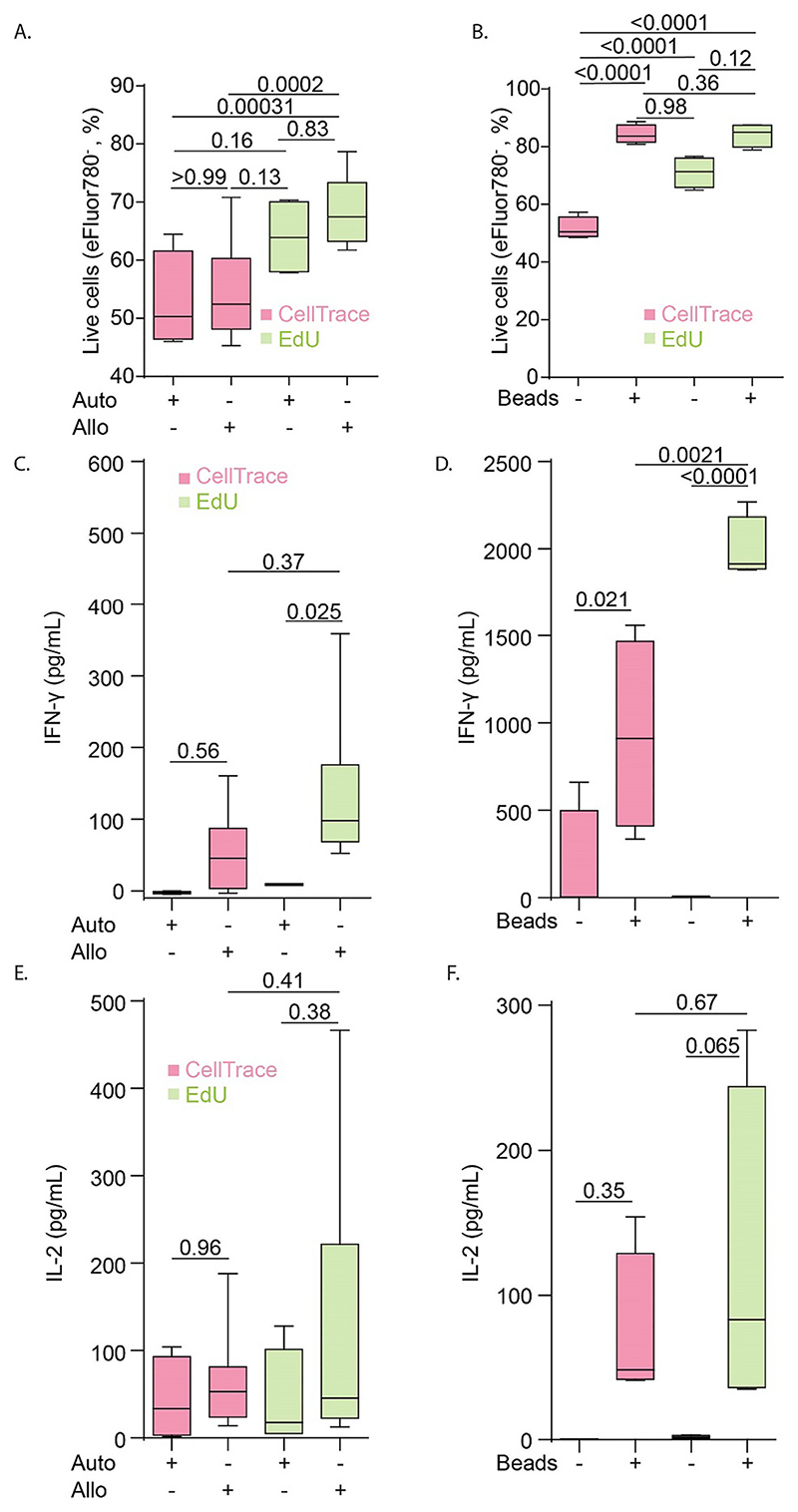
Viability assessment and cytokine detection in experiments with CellTrace and EdU. A-B. Quantification of cell viability detected for the MLR with autologous (Auto) or allogenic (Allo) pairs of PBLs and moDCs (A.), and for the anti-CD28 anti-CD3 bead-activated PBLs (+/-Beads) (B.) for the CellTrace (pink) and EdU (green) experiments. C. IFN-γ detection in supernatants from the MLR-experiments with CellTrace or EdU. D. Same as panel C, but for bead-stimulated PBLs. E-F. Same as panels C—D but for IL-2 production. All experiments were performed with cells isolated from 4 donors. Box plots represent mean, min and max values. *P*-values were evaluated with one-way ANOVA followed by Sidak’s post-hoc test. (For interpretation of the references to colour in this figure legend, the reader is referred to the web version of this article.)

Cells and supernatants from both experiments were collected for flow cytometry and ELISA analysis. Flow cytometry analysis of the viability probe eFluor780 (Figs. 1B and 2B) showed that the EdU protocol resulted in substantially less toxicity in the MLR and led to a 10-20% higher percentage of viable cells compared to the CellTrace-based protocol (Fig. 3A). Longer incubation times with EdU resulted in decreased viability, albeit not significant (Fig. Sup. 1). CellTrace also affected T cell viability in absence of stimulation, however, stimulation with anti-CD3 and anti-CD28-beads could rescue this viability (Fig. 3B). Thus, the cytotoxicity of CellTrace is especially pronounced in unstimulated T cells, and T cell activation prevents this loss of viability, likely by providing survival signaling (Vella et al., 1998) and/or because CellTrace becomes diluted in the daughter cells.

### 3.2 EdU facilitates sensitive detection of cytokine production

As other readouts of T cell activation, the levels of the T cell activation cytokines IFN-γ (Fig. 3C-D) and IL-2 (Fig. 3E-F) in the supernatants were measured. Upon co-culturing of the PBLs with autologous moDCs, we did not detect IFN-γ (Fig. 3C), and only low IL-2 levels (Fig. 3E). In the conditions with allogenic moDCs, the cells from the EdU experiments produced on average 2-fold more IFN-γ in comparison to the CellTrace experiments (Fig. 3C), while IL-2 production in activated cells was comparable between CellTrace and EdU (Fig. 3E). In conditions where PBLs were stimulated with anti-CD3 anti-CD28 beads, IFN-γ levels were also significantly higher in the EdU condition (Fig. 3D). However, IL-2 production was only slightly elevated in EdU condition treated with beads compared to the CellTrace condition (Fig. 3F). We also performed a combination of both CellTrace (added on day 0 of the T cell incubation) and EdU (added on day 4) treatment of cells, which showed that this combined treatment affected both IFN-γ and IL-2 cytokine production similarly to CellTrace alone (Fig. Sup. 2).

### 3.3 EdU-based protocol is more sensitive for detecting cell proliferation

**Fig. 4:**
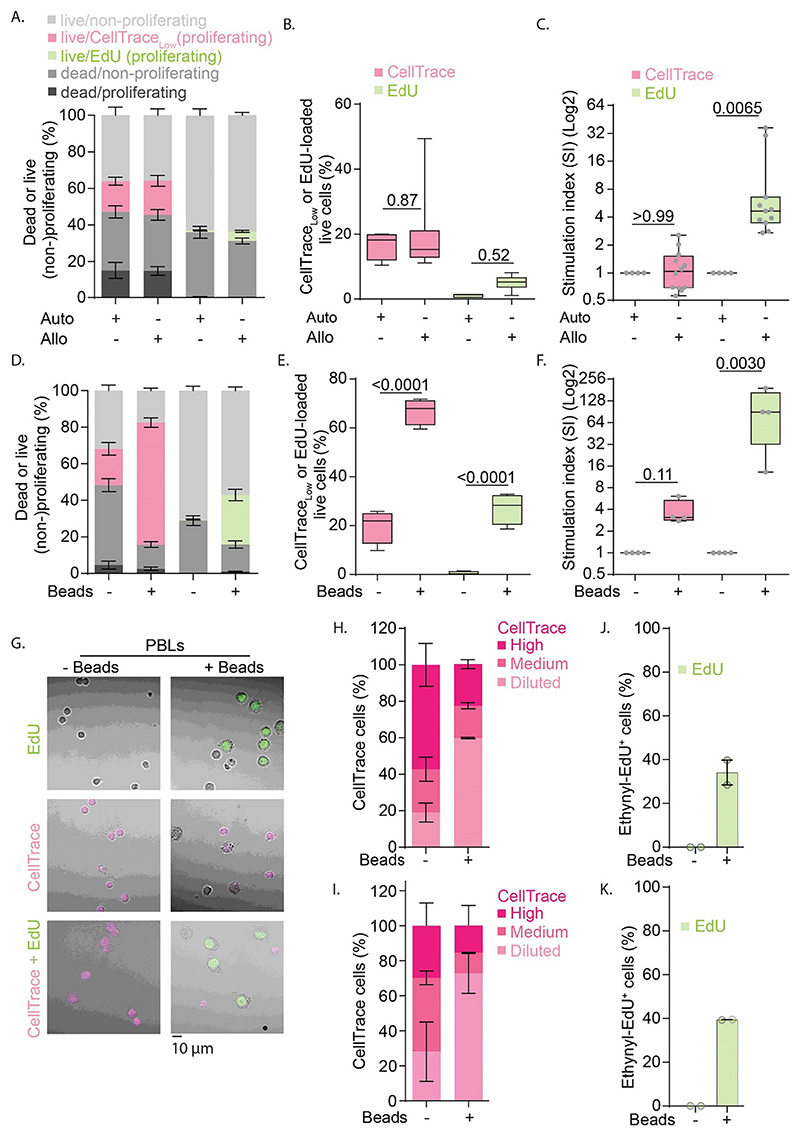
Assessment of PBL proliferation in experiments with CellTrace and EdU. A. Quantification of live, dead and proliferating and non-proliferating cells detected for MLR with autologous (Auto) or allogenic (Allo) pairs of PBLs and moDCs with CellTrace (pink) and EdU (green). B. Quantification of live proliferated cells. C. Normalization of MLR shown in panel B to the autologous conditions for each individual donor. D—F. Same as panels A-C, but now for PBLs stimulated with anti-CD3 anti-CD28 beads (+/-Beads). G. Representative confocal images of bead-stimulated PBLs. Cells were labeled with EdU (green), CellTrace (pink), or both. Scale bar, 10 μm. HK—. Quantification of fluorescence signals from panel G for experiments with only CellTrace (H.), only EdU (J.) and both (IK—.). For the CellTrace fluorescence (panels H and I), the signals were differentiated with manual thresholds in three categories: Diluted, Medium, and High. Experiments were performed with cells isolated from either 4 donors (panels A-F) or two donors (G-K). All box plots represent mean, min and max values. Bar graphs represent mean ± SEM. *P*-values were evaluated with one-way ANOVA (B, E) followed by Sidak’s post-hoc test or Kruskal-Wallis test on normalized data (C, F). For confocal imaging ~180 cells per condition were analyzed. (For interpretation of the references to colour in this figure legend, the reader is referred to the web version of this article.)

In the MLR experiments with CellTrace, only about 15% of the PBLs proliferated as seen from a reduced signal of CellTrace (Figs. 1B and 4A-B). However, the fraction of PBLs that divided did not differ between the co-cultures with allogenic or autologous moDCs (Figs. 1B and 4B). Normalizing the data to the autologous condition, to account for variables such as contaminations with non-lymphoid cells, the so-called stimulation index (SI) (Yu et al., 2009), also showed no differences in cell proliferation between the autologous and allogenic conditions (Fig. 4C). While it might be inferred from this that an MLR might not be a sufficient stimulant for T cell proliferation, the EdU experiments showed that this is not the case. Even though EdU only incorporated in about 5% of the PBLs during the final 2 h of the MLR with allogenic moDCs, this population was 3-30-fold higher (depending on the donor PBL-moDC combinations in the MLR) than with autologous moDCs (Fig. 4A-C). Similar cell division was observed for different azide functionalized dyes (Fig. Sup. 3). The experiments with the anti-CD3 anti-CD38 beads also showed that the EdU assay facilitated more sensitive detection of T cell proliferation. Although the CellTrace experiments showed a ~3-fold increased proliferation fraction with the beads compared to without beads, this increase was smaller than the ~90-fold increase in the fraction of EdU-incorporating cells (Fig. 4D-F). The finding that the percentages (Fig. 4B, E) and absolute numbers (Fig. Sup. 4) of proliferating cells in the unstimulated conditions (e.g., MLR auto or –Beads) with CellTrace are higher than with EdU (~2700 vs. ~250 in MLR auto; and ~650 vs. ~60 in –Beads), points at a higher background proliferation due to CellTrace.

In the CellTrace condition, the percentages of living non-proliferating cells are lower compared to the EdU condition (Fig. 4A, D). This is also reflected in the absolute numbers (Fig. Sup. 4). This difference can be explained partly, but not fully, by a higher number of cells maturing into proliferating cells in the CellTrace condition. For example, the number of proliferating cells in the autologous CellTrace experiments (mean ~ 2700) is much lower than the difference between the number of non-proliferating cells between CellTrace and EdU (~6500 vs. ~16,900 respectively, creating a difference of ~10,400 cells). These findings again indicate that CellTrace is toxic to non-proliferating cells. Indeed, the dead cells in the CellTrace condition are mostly positive for CellTrace (i.e., non-proliferating) (Fig. 4A, D). Similar conclusions cannot be drawn for the EdU condition, as proliferating cells that die before the 2 h of incubation with EdU will not be recognized as proliferating. It should be kept in mind that only the cells in the S-phase of the cell cycle are labeled with EdU, which could lead to less cells being recognized as proliferating compared to in the CellTrace condition. This too could contribute to the lower numbers of living cells that are recognized as non-proliferating in the CellTrace condition.

Additionally, we activated PBLs with anti-CD3 anti-CD38 beads, but now used confocal microscopy as another method for identifying T cell division (Fig. 4G). For the EdU protocol, we identified ~40% of proliferating (i.e., EdU-positive) cells (Fig. 4J-K), similar to the flow cytometry results (Fig. 4E). For the CellTrace experiments, we applied manual thresholds to separate the cells into three categories based on their CellTrace fluorescence intensities (Fig. 4H-I). Since we observed an increase in the fractions of cells with low CellTrace signals upon activation with the beads, we conclude that T cell division can also be inferred from microscopy images using CellTrace. However, in contrast to EdU, this analysis is based on arbitrary thresholding of the fluorescence, and individual proliferating cells can therefore not be identified with high certainty from the microscopy images.

### 3.4 EdU facilitates robust detection of T cell activation markers

**Fig. 5:**
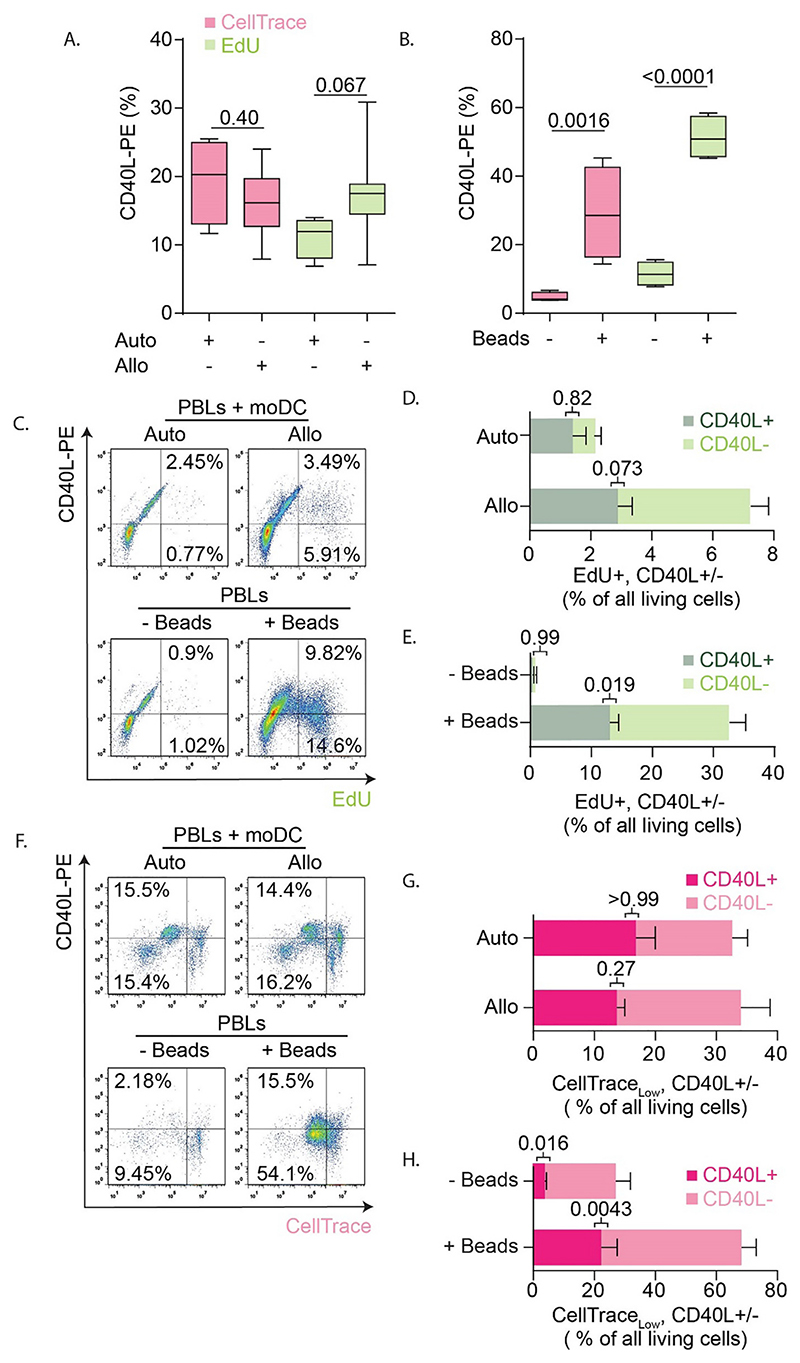
Expression of T cell activation marker CD40L in experiments with CellTrace and EdU. A-B. Quantification of CD40L expression for the MLR with autologous (Auto) or allogenic (Allo) pairs of PBLs and moDCs (A.), and for the anti-CD28 anti-CD3 bead-activated PBLs (+/-Beads) (B.) for the CellTrace (pink) and EdU (green) experiments. C. Scatter plot with flow cytometry gating strategy on the living cells from Fig. 2B with EdU (x-axis) and CD40L-PE (y-axis) for MLR (top row) and bead-stimulation (bottom row). D. Quantification of panel C for MLR reaction where the percentages of EdU^+^/CD40L^+^ and EdU^+^/CD40L^−^ T cells are quantified. E. Same as panel D, but now for bead-activated PBLs. F—H. Gating strategy and quantifications of CellTrace^low^/CD40L^+^ and CellTrace^low^/CD40L^−^ T cells for the CellTrace experiments. For all panels, experiments were performed with cells isolated from 4 donors. All box plots represent mean, min and max values. *P*-values were evaluated with one-way ANOVA followed by Sidak’s post-hoc test. (For interpretation of the references to colour in this figure legend, the reader is referred to the web version of this article.)

EdU can be combined with immunolabeling of cell surface antigens to measure T cell activation (Sun et al., 2012; Yu et al., 2009). We addressed how well T cell proliferation could be measured concomitantly with expression of CD40L. For the MLR experiments with CellTrace, we did not detect a higher CD40L expression in T cells incubated with allogenic moDCs versus autologous moDCs, as 15–20% of the T cells expressed CD40L regardless of whether they were incubated with autologous or allogenic moDCs (Figs. 1B and 5A). Although not significant, in the EdU experiments we observed a ~1.5-fold higher level of CD40L expression for the allogenic (~18%) compared to the autologous (~12%) pairs (Figs. 2B and 5A). In the CellTrace experiments with anti-CD3 and anti-CD28 beads, ~5% of the CD3^+^ T cells expressed CD40L and this increased to ~30% upon activation (Figs. 1B and 5B). For the EdU assay, the increase was stronger and ~50% of the bead-activated T cells expressed CD40L (Figs. 2B and 5B). The fact that not all activated cells are positive for CD40L can be explained by the transient nature of CD40L; the number of CD40L-positive cells is highest at 3–4 days after immunization (Van den Eertwegh et al., 1993).

Finally, we determined which fractions of living PBLs were both expressing CD40L and proliferating, as detected with EdU or CellTrace (Fig. 4B, E). With the EdU protocol, about one third of the dividing cells expressed CD40L in the stimulated samples (Fig. 5D, E). This was similar to our findings from the CellTrace experiments, where we also found that about a third of the cells that had divided (i.e., >2-fold reduced CellTrace intensity) expressed CD40L (Fig. 5G, H). Thus, about a third of proliferating T cells expressed CD40L regardless of the activation method and the assay for assessing cell proliferation.

## 4 Discussion

Evaluating antigen-specific T cell proliferation is an essential part of both fundamental and translational immunological research and is required for dissecting the cellular processes of immune-related diseases and diagnosis of antigen-specific immune responses. EdU is rapidly emerging as the technique of choice for measuring T cell proliferation, due to its ease of use and the short sample processing times (Alvarez et al., 2020; Poujol et al., 2014; Sun et al., 2016; Sun et al., 2012; Vibert and Thomas-Vaslin, 2017; Yu et al., 2009). However, a direct comparison of EdU with dilution-based T cell proliferation probes, the current standard in the field, was lacking. In this study, we performed a side-by-side comparison of EdU with CellTrace Far Red. Overall, we found that EdU is superior for determining T cell responsiveness, as it consistently showed less cytotoxicity in absence of T cell activation, increased levels of proliferation in PBLs, higher IFN-γ responses in the MLR, and higher CD40L expression. Thus, we observed larger differences between unstimulated and stimulated PBLs for almost all readouts, indicating that EdU is more sensitive than CellTrace Far Red for measuring T cell activation.

CFSE and related dilution-based probes such as CellTrace covalently bind to primary amines, and this is well known to affect the cell viability and function (Ten Brinke et al., 2017; Hasbold et al., 1999). This decrease in cell viability is not (only) caused by the long incubation times required for the CFSE assay, as cytotoxicity has already been observed in a T cell line immediately post-labeling (Filby et al., 2015). This finding suggests that the covalent modification of essential proteins by CFSE might render them inactive and induces cell death. Although CellTrace is generally less toxic than CFSE (Quah and Parish, 2012), it still affects the cell division program and reduces the number of proliferative cells (Filby et al., 2015). The cytotoxicity of CellTrace Far Red has been reported before (Tempany et al., 2018).

Overall, EdU labeled a smaller fraction of the PBLs than CellTrace. This is likely because only a minor fraction of the cells was in the S-phase of cell proliferation during the relatively short (2 h) incubation time with EdU. While prolonging the incubation time can indeed increase the number of EdU-positive cells (Sun et al., 2012), our data suggest that prolonged incubation times with EdU result in decreased viability. These data are in line with literature, as longer incubation with EdU has also been associated with increased cytotoxicity in various cell lines (Diermeier-Daucher et al., 2009; Haskins et al., 2020). Likely, the EdU alters the 3-dimensional structure of the DNA, which in turn alters the binding of DNA binding proteins and/or DNA proliferation resulting in cytotoxicity (Diermeier-Daucher et al., 2009). For the same reasons, prolonged incubation with EdU could be mutagenic (Haskins et al., 2020), which might limit its *in vivo* use. However, this might not be an issue, because the half-life of structurally related uridine analogs 5-ethyl-2’-deoxyuridine and BrdU are about one hour in mice (Cheraghali et al.,1995).

We show that in unstimulated conditions (i.e., autologous MLR and without beads), ~20% of the PBLs have a low signal for CellTrace, indicating that they proliferated despite the absence of stimulation. However, only low numbers of proliferating cells were observed with EdU. As about 20% of the non-stimulated CellTrace-labeled cells also expressed the T cell activation marker CD40L, this does not point at a labeling defect. Rather, CellTrace increases the background level of T cell activation. In addition, the reduced viability of CellTrace-labeled PBLs in absence of stimulation may have resulted in a bias in the data, as cell viability in the small fraction of (background) activated T cells could be preserved and they would hence be overrepresented. This CellTrace-induced death of non-activated cells may have particularly affected the MLR data, as allogenic moDCs only activate a small fraction of T cells. The lower toxicity of EdU is hence a major advantage for detecting proliferating T cells, as it does not suffer from the potential bias introduced by the killing of non-activated T cells with CellTrace.

Although the EdU assay does not require the harsh fixation and denaturation conditions of BrdU, it shares some of its other disadvantages. First, EdU does not allow the quantification of the rounds of division of the T cells, and appears to be somewhat cytotoxic for the long incubation times (24 h) needed for completion of a single round of cell division. In contrast, one of the advantages of dilution-based cell proliferation probes is the ability to distinguish single daughter generations, whereas this cannot be done with EdU. The EdU and CellTrace assays thus provide different information: whereas dilution-based T cell proliferation assays only reveal the number of cells that have divided, EdU only labels cells that are currently dividing. Second, the current EdU assay is an endpoint assay, because the bioorthogonal reaction requires fixation and cell membrane permeabilization. This can be overcome by pulse-chase experiments (Spada et al., 2005). An advantage of the EdU assay is that it could facilitate the capture of DNA from dividing cells using azide-conjugated beads, which would allow for the sequencing of DNA specifically from dividing T cells, similar to the antibody-based capture of cells with incorporated BrdU (Romar et al., 2016).

In short, we show that EdU is less toxic to non-stimulated T cells than CellTrace, and enables more sensitive detection of dividing cells. EdU allows for more sensitive discrimination of subtle IFN-γ responses, while the detection of IL-2 responses is comparable between EdU and CellTrace. Therefore, we conclude that for endpoint experiments, EdU is the assay of choice.

## Supplementary Material

Supplementary Material

## Data Availability

The data that support the findings of this study are openly available in Zenodo at https://zenodo.org/record/5788359#.YbxaVGjMI2w, DOI: 10.5281/zenodo.5788358
