# Supplementary material for: A sensitive and less cytotoxic assay for identification of proliferating T cells based on bioorthogonally-functionalized uridine analogue
Source: J Immunol Methods. Author manuscript; Available in PMC 2023 Jun 23. (PMC7614694; doi:10.1016/j.jim.2022.113228)
Supplement: Supplementary Material [file EMS177387-supplement-Supplementary_Material.docx]

**5. Supplementary information**

**5.1 Supplementary Figures**

***
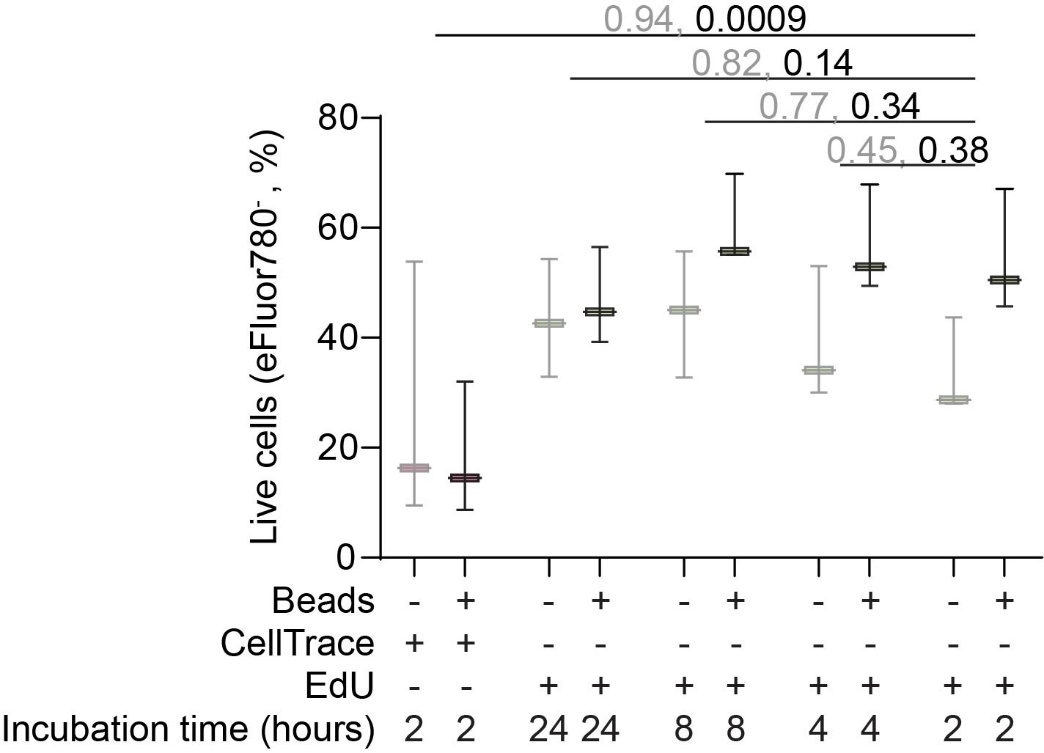
***

***Supplementary Figure 1. Viability assessment with longer incubation times with EdU.*** *Quantification of cell viability detected for anti-CD28 anti-CD3 bead-activated PBLs (-/+Beads). Conditions without beads are depicted in gray, where conditions with beads are depicted in black. Experiments were performed with cells isolated from 3 donors. All box plots represent mean, min and max values. P-values were evaluated with two-way ANOVA followed by Sidak’s post-hoc test.*

**
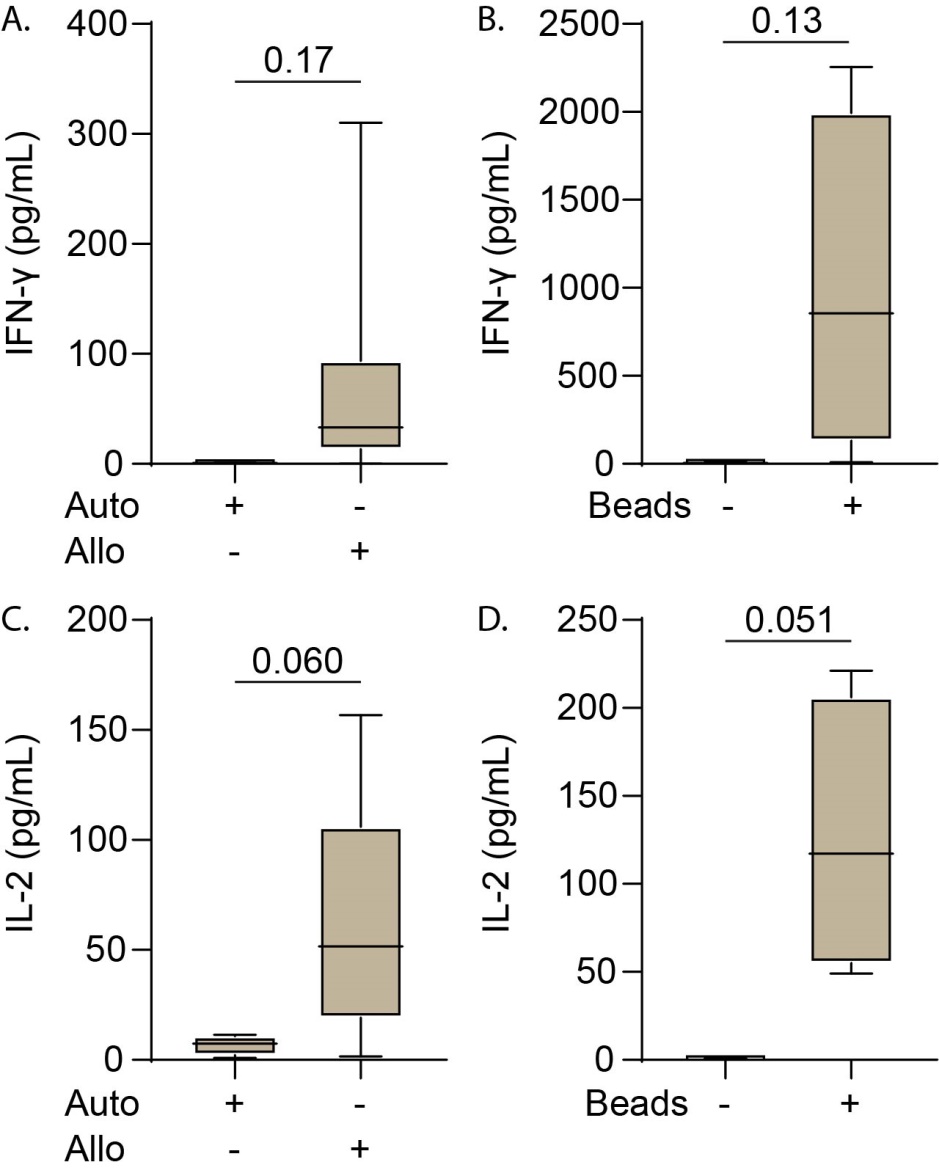
**

***Supplementary Figure 2. Cytokine production in experiments with combined CellTrace and EdU.*** ***A.*** *IFN-γ detection in supernatants from the MLR-experiments as in main figure 3, but now with a combination of CellTrace and EdU. P-values were evaluated with unpaired T test.* ***B.*** *Same as panel A, but for bead-stimulated PBLs. P-values were evaluated with paired T test.* ***C-D.*** *Same as panels A-B but for IL-2 production. All experiments were performed with cells isolated from 4 donors. Box plots represent mean, min and max values. NS: not significant.*

**
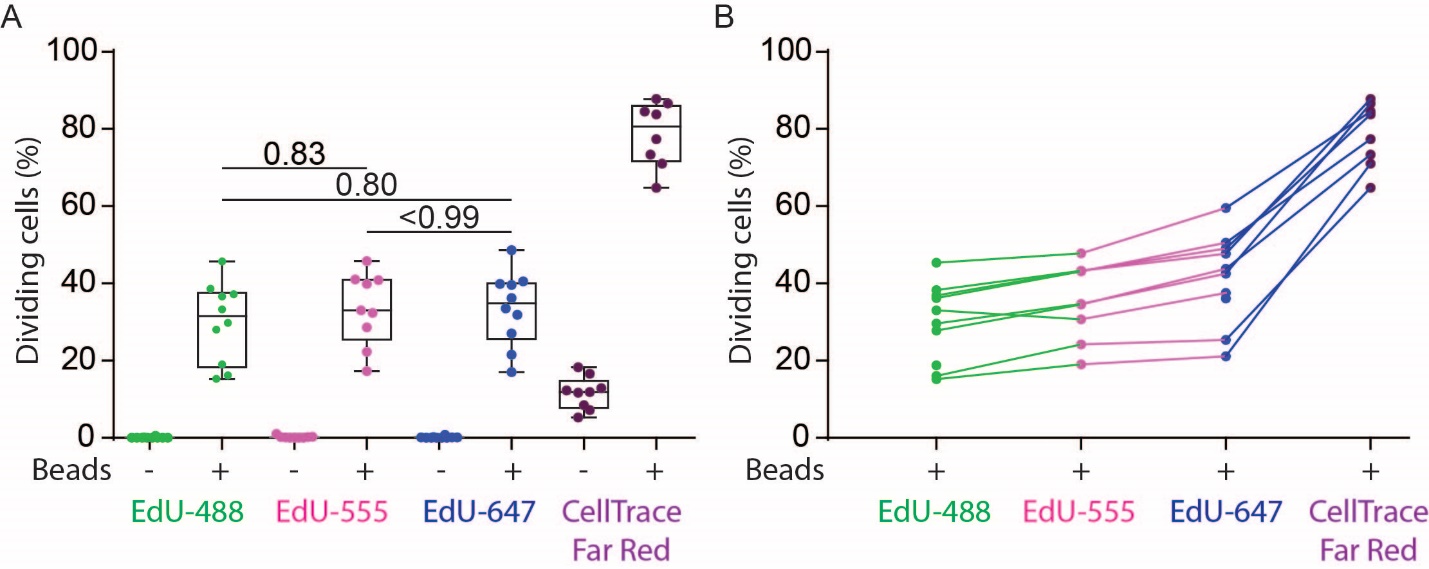
**

***Supplementary Figure 3. EdU assay with different azide-functionalized probes.*** *(****A.****) EdU proliferation assays with EdU488 (6-FAM azide alernative), EdU555 (5-TAMRA azide alternative) and EdU647 (Cy5 alternative) and CellTrace Far Red assay. PBLs are activated with anti-CD3 and anti-CD28 beads (+/-Beads). Experiments were performed with PBLs isolated from 10 donors. P-values were evaluated with one-way ANOVA followed by Sidak’s post-hoc test. Flow cytometry gating strategies used are shown in figure 1 (CellTrace) and 2 (EdU). (****B****) Bead-stimulated samples from panel A, but now different conditions of the same donor are connected.*


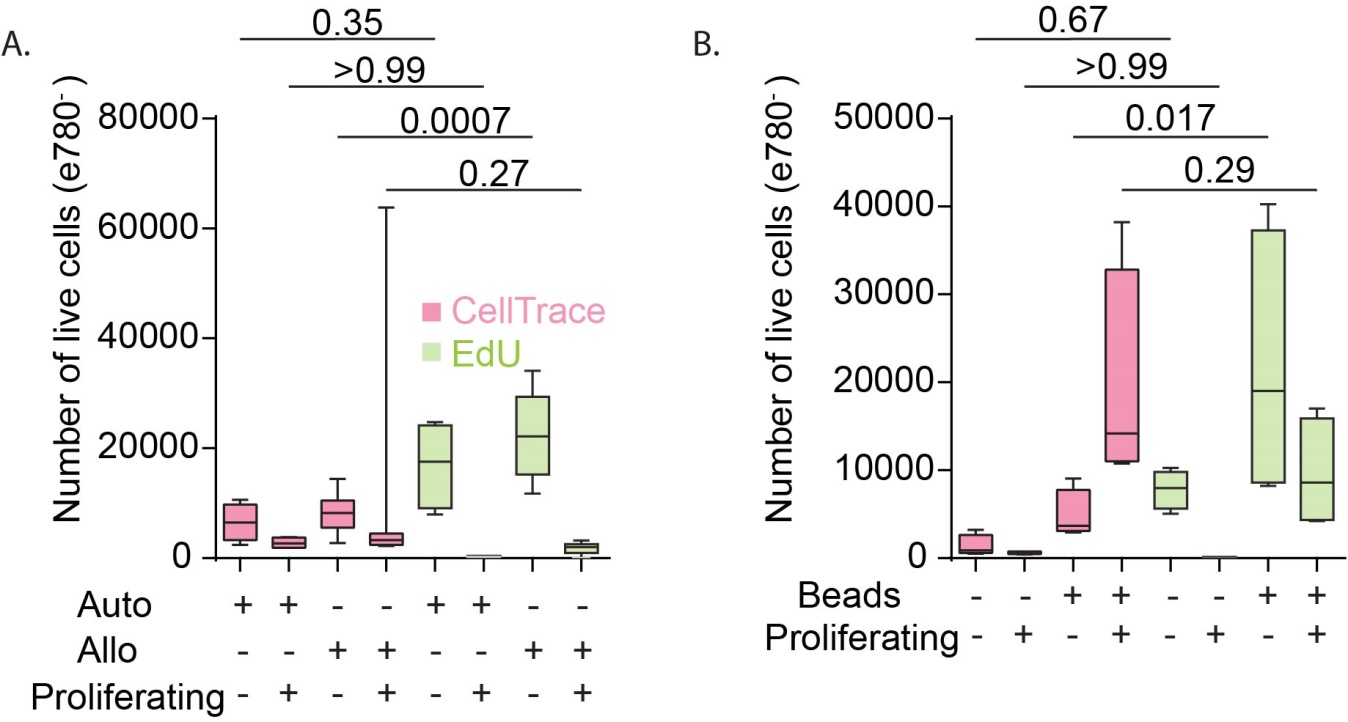


***Supplementary Figure 4****.* ***Absolute numbers of living cells in experiments with CellTrace and EdU.*** *Numbers of proliferating and non-proliferating living cells in the MLRs with autologous (Auto) or allogenic (Allo) pairs of PBLs and moDCs (****A.****), and in the anti-CD28 anti-CD3 bead-activated PBLs (+/-Beads) (****B.****) for the CellTrace (pink) and EdU (green) experiments. All experiments were performed with cells isolated from 4 donors. All box plots represent mean, min and max values. P-values were evaluated with one-way ANOVA followed by Sidak’s post-hoc test.*

**5.2 STAR methods table (Key resource table)**

| **REAGENT or RESOURCE** | **SOURCE** | **IDENTIFIER** |
| --- | --- | --- |
| **Antibodies** | | |
| Mouse anti-CD40L (CD154) (clone TRAP-1) PE | Beckman | Ref. IM2216U |
| Mouse anti-CD3 (clone UCHT1) APC | Invitrogen | Ref. 17-0038-42 |
| Mouse anti-CD3 (clone SK7) BV421 | BD Biosciences | Ref. 563798 |
|  |  |  |
| **Chemicals** | | |
| Paraformaldehyde | Electron Microscopy Sciences | Cat. no. 15710 |
| Phosphate buffer saline (PBS) | Gibco | Ref. 14190-144 |
| Bovine Serum fraction IV | Gibco | Ref. 15260-037 |
| Cell Trace | Invitrogen | Ref. C34564 |
| Antibiotic-Antimycotic Solution | Gibco | Ref. 15240-062 |
| Glutamine | Gibco | Ref 25030-149 |
| FBS | HyClone | 20200002 |
| Human Serum | Sigma-Aldrich Life Science | Product. no. H4522 |
| RPMI | Gibco | Cat. no. 21875034 |
| Ficol | STEMCELL Lyphoprep | 04-03-9391/02 |
| Poly-lysine | Thermo Fisher | Cat.no A3890401 |
| **Critical Commercial Assays** | | |
| Eterneon GREEN-Azide kit | Baseclick GmbH | Cat. no. BCK-TCell-FC488 |
| ClickTech EdU T Cell Proliferation kit 488 | Baseclick GmbH | Ref. CC06005 |
| CellTrace Far Red cell proliferation kit | Invitrogen | Cat. no. C34564 |
| Human IFN-γ uncoated ELISA kit | Thermo Fisher Invitrogen | Ref. 88-7316-88 |
| Human IL-2 uncoated ELISA kit | Thermo Fisher Invitrogen | Ref. 88-7025-88 |
| CD8 isolation kit |  |  |
| Dynabeads | Gibco | Ref. 11131D |
| Fixable Viability Dye eFluor 780 | eBioscience | Ref. 65-0865-14 |
|  |  |  |
| **Lab plasticware** | | |
| T75 Cell Culture Flask | Corning Costar | Ref 431464U |
| Controlled-rate cell freezing container for cryogenic vials (Mr. Frosty) | Corning CoolCell LX | CLS432001 |
| Cryo.S cryogenic vials | Greiner | Ref. 126263 |
| SepMate-50 tubes for density gradient centrifugation | SepMate | Ref. 85450 |
| High-affinity protein binding 96-well ELISA plates | ThermoFisher | Cat. no. 88-7316 |
| Ibidi µ-Slide 8 Well high Glass Bottom | Ibidi | Cat. no. 80807 |
|  |  |  |
| **Experimental Models: Primary Cells** | | |
| Human monocyte-derived dendritic cells (moDCs) | Blood donors | N/A |
| Peripheral blood lymphocytes (PBLs) | Blood donors | N/A |
|  |  |  |
| **Software and Algorithms** | | |
| GraphPad Prism 8 | GraphPad Software | https://www.graphpad.com/; RRID:  SCR_002798 |
| ImageJ software (Fiji v. 2.0.0-rc-  65/1.51w) | NIH | https://imagej.net/; RRID: SCR_003070 |
| FlowJo |  | https://www.flowjo.com/ |
| CytoFlex S | Beckman Coulter |  |
|  |  |  |
